# A New Microwave-Assisted Protocol for Cellulose Extraction from Eucalyptus and Pine Tree Wood Waste

**DOI:** 10.3390/polym16010020

**Published:** 2023-12-20

**Authors:** Silvia Vinhas, Mafalda Sarraguça, Tânia Moniz, Salette Reis, Maria Rangel

**Affiliations:** 1 LAQV, REQUIMTE, Departamento de Química e Bioquímica, Faculdade de Ciências, Universidade do Porto, Rua do Campo Alegre, s/n, 4169-007 Porto, Portugal; silvia.vinhas@fc.up.pt; 2 LAQV, REQUIMTE, Departamento de Ciências Químicas, Faculdade de Farmácia, Universidade do Porto, Rua de Jorge Viterbo Ferreira, 228, 4050-313 Porto, Portugal; mafalda.cruz@ff.up.pt (M.S.); shreis@ff.up.pt (S.R.); 3 LAQV, REQUIMTE, Instituto de Ciências Biomédicas de Abel Salazar, Universidade do Porto, Rua de Jorge Viterbo Ferreira, 228, 4050-313 Porto, Portugal; mrangel@icbas.up.pt

**Keywords:** microwave-assisted extraction, cellulose extraction, hemicellulose, lignin, wood wastes, sustainability

## Abstract

An enormous interest in the development of efficient protocols for cellulose extraction has been demonstrated in the last few years, although usually based on non-sustainable chemical and thermal approaches. In this work, we propose a new and more sustainable method for cellulose extraction from eucalyptus and pine tree wood waste products exclusively performed using microwave-assisted radiation. The methodology includes three main steps: (i) alkaline treatment; (ii) bleaching I, using H_2_O_2_; and (iii) bleaching II, an acidic treatment. Samples obtained in each step were characterized by Fourier-transform Infrared (FTIR) spectroscopy, powder X-ray diffraction (PXRD), thermogravimetric analysis (TGA), and differential scanning calorimetry (DSC). The results were compared with the structural and thermal profile of the starting materials, a commercially available microcrystalline cellulose and with an industrial paper pulp sample. Results confirmed that for both types of wood wastes, cellulose was retained during the extraction procedures and that the removal of hemicellulose and lignin was mainly achieved in the last step, as seen by the FTIR spectra and TGA curves. The developed protocol is innovative, as it constitutes an easy and quick approach for extracting cellulose from eucalyptus and pine tree wood waste. Mild chemical and thermal conditions are used during the three extraction steps (microwave irradiation, aqueous solutions, maximum of 120 °C in a total of 3 h). Moreover, environmentally friendly purification steps are applied based on the use of water and ethanol. This approach offers the possibility of a future scale-up study to potentially apply the developed protocol to the extraction of cellulose on an industrial scale.

## 1. Introduction

Cellulose, which has a structure consisting of β-1,4-linked anhydro-D-glucose units, is a naturally occurring polymer in plants and the most abundant source of lignocellulosic biomass [1,2]. This biopolymer is a valuable resource for many types of applications due to its low cost, high abundance, great biocompatibility, biodegradability, and non-toxic nature [1,3]. Thus, many efforts have been made to develop efficient extraction methodologies in order to obtain cellulose for further transformation into high-value products such as viscose rayon, cellulose nitrate, cellulose acetate, and nanocrystalline cellulose (reviewed in [4]). However, the extraction of this polymer from raw materials with a high degree of purity represents a challenge due to the presence of other major components in the biomass, mainly lignin [5] and hemicellulose [6]. The lignin content depends on the natural source, ranging from approximately 10 to 25% [7], while hemicellulose is present within the interval of 20–40% [8].

Several chemical and thermal methods have been reported for the removal of these components, such as organo-solvent extraction and enzymatic hydrolysis. Even though these methods may represent easy and low-cost options, they are time-consuming, have poor extraction yield, and use a great volume of solvent, most of a non-benign nature, as well as high temperature and pressure conditions, as referred to in [2,3,4].

Microwave-assisted extraction (MAE) methods have been explored in the last decades to develop more sustainable, faster, and more efficient approaches to cellulose extraction [3]. The use of microwave radiation in the heating process offers many advantages in extraction processes, including lower reaction time, the use of high dielectric constant/polar solvents to improve energy absorption, and lower solvent consumption [9,10]. Particularly for plant subtracts, MAE procedures can improve cell wall disruption, allowing for better extraction of the components such as lignin and hemicellulose [2].

The most rudimentary studies were performed considering the extraction of cellulose using simple domestic microwave equipment, eventually in combination with conventional heating methods [3,11]. Other investigations employed more advanced laboratory devices in which the developed MAE methodologies for cellulose extraction considered single vessel (monomode) microwave equipment, usually using close vessels to increase the range of temperature and pressure. Among these approaches, several were exclusively performed using microwave radiation [12] or in combination with traditional heating methods [13,14].

The use of monomode equipment can limit the scalability of the process due to the reduced volume of solvents that can be used (mL scale), in comparison to the alternative multimode apparatus that offers the possibility of using volumes up to a few liters [15]. Considering this, studies have been reported such as the work developed by Kusumattaqiin and Chonkaew [16], in which the authors proposed a method for cellulose extraction from cotton wool using the MAE technique with multimode equipment. Other reports have described similar approaches considering different cellulose sources in combination with conventional heating methods [2] or other techniques such as ultrasound [17].

In addition to several types of extraction methods, different cellulose sources can be considered, mainly comprising plants or algae feedstocks [1,2,13]. Many studies have focused on sustainable methods for the valorization of plants, particularly from fruits [12] as well as byproducts and waste generated from fruit processing [3,18]. Among other sources, wood waste from trees has been largely explored as a valuable source for the extraction of this biopolymer, as evidenced by many reports in the literature [5,6,19,20,21]. From the large variety of wood that can be used for cellulose extraction, numerous studies have reported eucalyptus and pine trees as interesting biomass feedstocks [22,23,24]. A number of the referenced studies include MAE techniques to obtain the desired polymer, such as the work developed by Hou et al. [4], in which eucalyptus wood was considered as a natural source for cellulose extraction and MAE was included as part of the process. In another study, pine and eucalyptus wood were selected as cellulose sources, and the proposed extraction process involved the use of microwave heating methods together with ionic liquids to improve the efficacy of the extraction process [21]. However, this methodology included a less sustainable purification process, as the authors considered the use of non-environmentally friendly solvents, specifically dimethyl sulfoxide (DMSO).

The use of such cellulose sources can contribute to better use of wood feedstocks usually considered to be waste.

Eucalyptus and pine tree wood samples were selected as a lignocellulosic source in this study, as they have high cellulose content and are present in great abundance in Portugal. Pine and eucalyptus tree woods differs in nature and composition, with the former considered a softwood [19] and the latter classified as a hardwood [25]. This aspect can be relevant for the efficiency of the extraction process, as it has been reported that softwoods are harder to delignify than hardwoods in general [26,27].

Thus, the main aim of the present work consists of the evaluation of the applicability of a newly developed approach to extract cellulose from eucalyptus and pine tree wood samples. An additional objective of this work to verify whether the protocol is valid for the two distinct categories of wood (soft and hard) considered herein.

To accomplish these objectives, a comparison of the extracts obtained in each step of the methodology has been performed while considering structural and thermal analysis of the samples from both sources and comparing them with commercially available microcrystalline cellulose and an industrial paper pulp sample.

The extraction protocol proposed herein is one of the very few to consider both softwoods and hardwoods using mild extraction conditions and benign purification solvents. Moreover, the methodology exclusively uses the MAE technique in a multimodal apparatus, offering great advantages for a future scale-up study to potentially apply the developed method to the extraction of cellulose on an industrial scale.

## 2. Materials and Methods

### 2.1. Materials and Instrumentation

NaOH, H_2_O_2_ (30% *w*/*w*, Perdrogen), H_2_SO_4_, and 1-Ethyl-3-methylimidazolium chloride were acquired from Sigma-Aldrich (St. Louis, MO, USA). Wood samples (small branches from eucalyptus and pine trees) were collected from urban gardens in the Porto region (Gondomar, Portugal). Industrial paper pulp samples were helpfully provided by a paper company.

Extractions were processed using multi-vessel MARS 6™ microwave-heating equipment (CEM Corporation, Matthews, NC, Canada) using glass containers. Each extraction step was performed considering two independent replicates. Fourier Transform Infrared Spectroscopy (FTIR) spectra were recorded in a PerkinElmer Frontier (Beaconsfield, UK) equipped with an attenuated total reflectance (ATR) accessory with a zinc selenite crystal. The ATR accessory has a pressure arm to control the applied force and reduce sample-to-sample variability. Thermogravimetric analysis (TGA) was performed at FCUP|DQB-Lab&Services using a Hitachi STA7200RV as equipment (Tokyo, Japan). Differential Scanning Calorimetry (DSC) studies were performed on DSC 200 F3 Maia equipment with an automatic sample changer (Netzsch, Selb, Germany). Powder X-ray Diffraction studies were performed at UniNova, Nova School of Science and Technology, Lisbon, Portugal, using Malvern Panalytical X’Pert PRO MPD equipment (Malvern, UK).

### 2.2. Wood Samples Pre-Treatment

**Figure 1:**
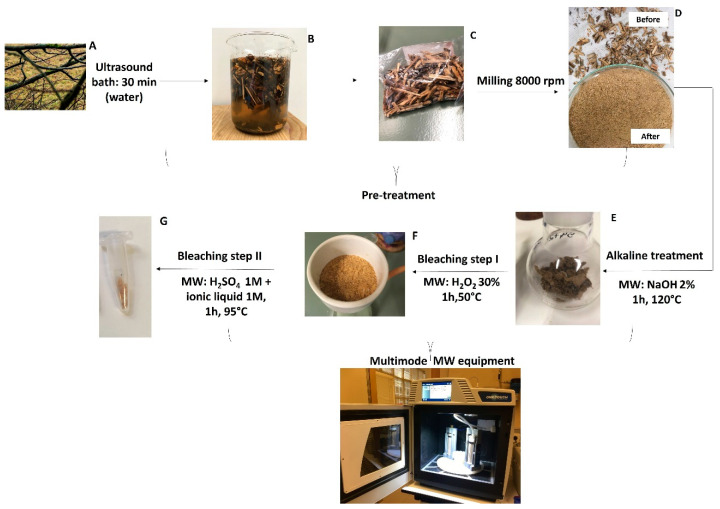
Experimental procedure for the microwave-assisted extraction of cellulose from eucalyptus and pine tree wood waste samples: (**A**) starting materials; (**B**) washed wood samples; (**C**) dried wood samples after washing; (**D**) powdered wood; (**E**) extraction fraction after the alkaline treatment; (**F**) extraction fraction after bleaching I; and (**G**) extraction fraction after bleaching II.

Wood samples (Figure 1A) were cut into small pieces of approximately 0.5 cm in length and then washed in deionized water and subjected to an ultrasonic bath for 1 h to remove soil and other residues (Figure 1B). After the wash pre-treatment, samples were left to dry (Figure 1C) in an oven at 50 °C, overnight. The dried wood samples were then milled in an Ultra Centrifugal Mill ZM 200 processor operating at 8000 rpm (Retsch, Haan, Germany). Pulverized samples (Figure 1D) were then considered for the alkaline treatment.

### 2.3. Alkaline Treatment

Pulverized eucalyptus and pine wood samples (1 g) were first treated in an alkaline aqueous solution of NaOH (10 mL, 2% *w*/*v*); the reaction was carried out for 1 h at 120 °C with a maximum power of 600 W using the multimode microwave equipment (glass containers) [14]. At the end of the reaction, the samples were filtrated; the solid part was recovered, and the supernatant (a black liquor) was rejected. The solid was washed with water and ethanol until the remaining solution became clean with a neutral pH (pH indicator strips pH 0–14, Merck, St. Louis, MO, USA). Samples were then dried overnight in a drying vacuum pump at 65 °C and 35 mBar and stored in a desiccator at room temperature until needed.

### 2.4. Bleaching I

To perform the first step of the bleaching treatment, 250 mg of each sample from the alkaline treatment was mixed with 10 mL of the bleaching agent (H_2_O_2_, 30% *w*/*w*) [2]. The reaction mixture was subjected to microwave radiation at 50 °C for 1 h using a maximum power of 600 W. At the end of the reaction, the resultant solid material was filtered under vacuum and the material was washed four times with water and ethanol. The product was then dried and stored as described for the alkaline step of the reaction.

### 2.5. Bleaching II

The second step of the bleaching process consisted of the reaction of 200 mg of each sample from bleaching I with 10 mL of an aqueous solution of H_2_SO_4_ (1 M) and an ionic liquid, 1-Ethyl-3-methylimidazolium chloride (1 M, 10% *v*/*v*) [13]. The reaction was placed in the glass vessel for the microwave reaction, which occurred at 95 °C for 1 h with a maximum power of 600 W. Afterwards, the products were washed as described in the previous steps and dried under similar conditions.

### 2.6. Characterization of the Extracted Samples

#### 2.6.1. Fourier Transform Infrared Spectroscopy (FTIR)

The FTIR spectra of the wood samples subjected to the different chemical treatments were obtained placing the samples directly in the ATR accessory. Spectra were recorded between 4000 cm^−1^ and 600 cm^−1^ with a resolution of 4 cm^−1^ and 16 co-added scans. The background was obtained with the ATR accessory empty. Microcrystalline cellulose, industrial paper pulp, and the starting materials from both eucalyptus and pine tree wood were analyzed as controls. The two experimental replicates obtained in each extraction step were analyzed; for each sample, three spectra were recorded, and the average spectrum was calculated. The average spectrum was then used for further analysis.

- Principal Component Analysis (PCA)

For the PCA analysis, the FTIR spectra were preprocessed with standard normal variate (SNV) and mean-centered (MNCN). PCA was performed using Matlab 2022a software version R2022a Update 5 (9.12.0.2039608) (MathWorks, Natick, MA, USA) and PLS Toolbox version 9.2.1 (2023) (Eigenvector Research Inc., Manson, WA, USA).

#### 2.6.2. Powder X-ray Diffraction (PXRD)

Powder X-ray diffractograms were obtained considering the following measurement conditions: Theta angles from 5° to 70°; step size of 0.033° 2Theta; counts/step* = 45 s (using a 1D detector); and total scan time of 10 min. The instrumental settings were: tube settings of 45 KV and 40 mA; Divergent Slit −1/2°; AntiScatter Slit- 1°; and a nickel filter on the detector side. Samples were mounted on an Si-0 background holder to avoid background signals on the diffractograms. The same control samples described in Section 2.6.1 were considered for these studies. One representative sample of each extraction step was analyzed.

#### 2.6.3. Thermogravimetric Analysis (TGA)

For the TGA analysis, the amount of sample used for each measurement was approximately 20 mg. All measurements were carried out under a nitrogen atmosphere with a flow rate of 200 mL·min^−1^ by heating the material from 20 to 800 °C at a heating rate of 10 °C·min^−1^. The same control samples described in the previous sections were considered for these studies. One representative sample of each extraction step was analyzed.

#### 2.6.4. Differential Scanning Calorimetry (DSC)

DSC thermograms of wood samples subjected to the different chemical treatments, the microcrystalline cellulose, the industrial paper pulp, and the starting materials from both trees were obtained. Accurately weighed samples (1–2 mg) were poured into aluminum pans and hermetically sealed. The samples were scanned from 30 to 450 °C at 10 °C·min^−1^. Nitrogen gas was used as purging gas at 50 mL·min^−1^. The onset temperature was calculated using NETZSCH Proteus^®^ Software–Thermal Analysis–Version 6.1 software. One representative sample of each extraction step was analyzed.

## 3. Results and Discussion

### 3.1. Development of the Protocol for Cellulose Extraction from Wood Samples

In the present work, eucalyptus and pine tree wood wastes were used as starting materials to implement a new protocol for cellulose extraction using a simple, and sustainable method. The applicability of the developed approach was evaluated by qualitative analysis of the obtained samples in each reaction step based on the information acquired from the spectroscopic and thermal characterization techniques. The analysis herein performed considered a comparison between the structural and thermal features of the obtained products and those of industrial paper pulp and commercial microcrystalline cellulose.

The new methodology presented in this work includes a pre-treatment followed by three main steps: alkaline treatment (A); a first bleaching step (B1); and a second bleaching step (B2).

Pre-treatment was employed to remove soil and other residues present in the wood samples. As can be seen in Figure 1B, samples were cleaned by washing in an ultrasound bath, with the residues retained in the water, as could be visualized directly. The pre-treated samples were then dried and milled (Figure 1C and Figure 1D, respectively) for the following step.

The first step consists of an alkaline treatment (Figure 1E), in which an alkaline solution of NaOH 2% (*v*/*v*) [12,13,14] is used to target the removal of hemicellulose, which is commonly present in wood samples [2]. The second step (Figure 1F), bleaching I, comprises the use of H_2_O_2_, which is considered a chlorine-free green bleaching agent [12], with the aim of delignifying the samples [13]. The last step (Figure 1E), bleaching II, is based on literature reports which have considered acid treatments [11,14,28] as an additional stage to improve lignin removal, particularly including those involving the use of an aqueous solution of H_2_SO_4_ [3,13,28] and an ionic liquid [13]. Of note, ionic liquids have drawn much attention in cellulose extraction processes and have been referred to by different authors [3,11,29,30]. This is because their cation and anion components can disrupt the linkages in cellulosic biomass, thereby enhancing the removal of lignin and hemicellulose. In particular, Hou and colleagues [4] demonstrated that a combination of microwave radiation and ionic liquids can improve cellulose extraction from eucalyptus wood. For this reason, the use of this type of solvent was considered in the methodology developed herein. However, in the aforementioned work the authors considered a additional step comprising of enzymatic hydrolysis for the obtention of cellulose in the subsequent extraction steps, which is not considered in our study.

The protocol established herein differs from others in the literature in that it considers easy purification steps and consists only of the filtration of samples using a benign mixture of solvents, particularly water/ethanol, instead of less environmentally friendly solvents such as the DMSO used in the previous methodology developed for cellulose extraction from eucalyptus and pine tree samples [21].

To evaluate the success of the applied treatments, the extraction fractions from each step were characterized by different techniques, as previously described.

### 3.2. Characterization of the Extracted Products

#### 3.2.1. Fourier Transform Infrared Spectroscopy

**Figure 2:**
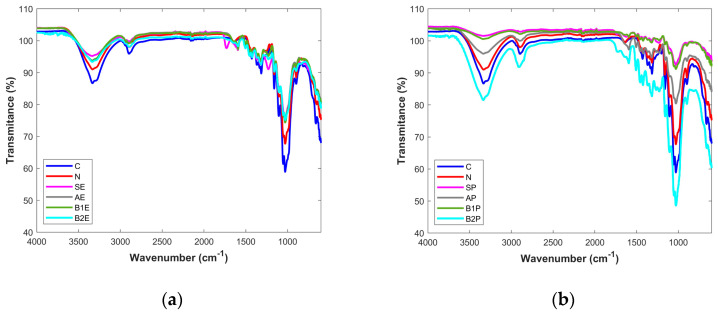
Spectra from (**a**) eucalyptus and (**b**) pine tree samples. C—Microcrystalline cellulose; N—pulp paper; SE (P)—starting materials; AE(P)—sample after the alkaline treatment; B1E(P)—samples after bleaching I; B2E(P)—samples after bleaching II.

The extracted products obtained in each reaction step were analyzed by FTIR spectroscopy and compared to the starting materials (pretreated wood, SE, SP), as well as with commercially available microcrystalline cellulose (C) and industrial pulp paper (N). Figure 2a,b summarizes the results obtained for eucalyptus and pine tree wood samples, respectively.

The FTIR spectrum of the industrial pulp paper sample (N) shows great similarity with the spectrum obtained for the pure cellulose (C), revealing the characteristic cellulosic peaks at 1160 cm^−1^, 1050 cm^−1^, 1030 cm^−1^, and 895 cm^−1^ [12,13,14]. Regarding the starting materials from both types of wood (SE, SP), it was possible to conclude that other bands are present in addition to the characteristic bands from cellulose polymer; in particular, a band at 1230 cm^−1^, a set of bands from 1320–1600 cm^−1^, and two others appearing at 1620 cm^−1^ and 1720 cm^−1^ form the other wood components, mainly hemicellulose and lignin. After alkaline treatment, for both types of wood (AE and AP) it can be seen in the spectra that the band at 1720 cm^−1^ almost disappears. Peaks appearing at 1620 cm^−1^ and 1720 cm^−1^ become extinct, and a decrease of bands between 1320–1600 cm^−1^ can be verified as well.

There are many reports in the literature suggesting that the prominent peaks at 1732 cm^−1^ and 1235 cm^−1^ are assigned to the C=O linkage of the acetyl and uronic ester groups present on hemicellulose structure or to the ester linkage of the carboxyl groups of lignin and/or hemicellulose [14,20,28]. Therefore, a decrease in the wavelength of the peaks at 1720 cm^−1^ and 1230 cm^−1^ suggests the removal of hemicellulose after the alkaline treatment. Another study reported that the vibration at 1628 cm^−1^ is due to the presence of proteins [18] and that this band was totally removed after alkaline treatment. Therefore, it is plausible to conclude that this extraction step was efficient in removing the protein content present in the wood samples. According to the literature reports, lignin presents characteristic peaks in the range of 1450 to 1600 cm^−1^, which are due to the aromatic skeletal vibration [12,14,20,28]. Because the bands in this range decreased after the first step of the chemical treatment applied to the wood samples, it is possible to suggest that lignin was partially removed from the starting materials.

The subsequent chemical treatment steps led to slight differences in the characteristics of all peaks in the FTIR spectra.

**Figure 3:**
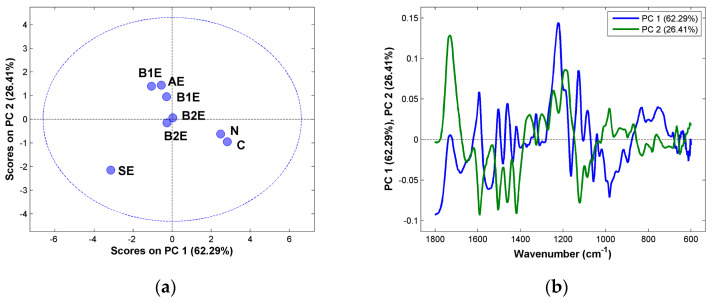

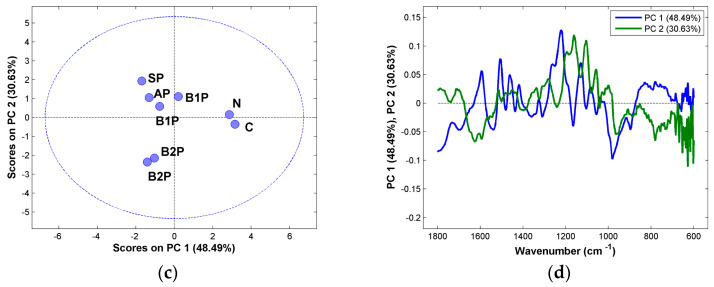
PCA performed with the SNV preprocessed FTIR spectra from the (**a**,**b**) eucalyptus and (**c**,**d**) pine tree samples between 1800 cm^−1^ and 600 cm^−1^. Score plots (**a**,**c**); loading plots (**b**,**d**).

For a more detailed examination of the data, Principal Component Analysis (PCA) was performed in order to obtain deeper insight into the effect of each chemical treatment (Figure 3). Before the PCA, the FTIR spectra were preprocessed with SNV and analyzed between the range 1800 cm^−1^ and 600 cm^−1^. The preprocessing was performed to remove certain effects (physical and instrumental) that were not related to the chemical process.

In the PCA score plot for the eucalyptus samples (Figure 3a), microcrystalline cellulose sample (C) and the paper pulp sample (N) are separated in the first component from the rest of the samples, in particular from the starting material (SE). This separation is an indication of the increased degree of purity in terms of cellulose content from the starting material (SE) until the microcrystalline cellulose (C). In the second component, the samples are separated according to the amount of hemicellulose. The sample after the alkaline treatment (AE) has similar bands to hemicellulose and the ones after bleaching I (B1E); however, the samples after the second bleaching are more similar to pure cellulose, with a lower hemicellulose content. These conclusions are confirmed by the analysis of the loadings (Figure 3b). It can be seen that, for both of the first components, the bands due to cellulose (1160 cm^−1^, 1050 cm^−1^, 1030 cm^−1^) are responsible for the separation seen in the score plot. For the second component, the bands due to hemicellulose (1320–1600 cm^−1^, 1620–1720 cm^−1^) are responsible for the separation seen in the score plot.

A similar analysis can be performed for the pine samples. The differences in the first component of the score plot (Figure 3c) are due to differences in the degree of purity in the cellulose content, The removal of hemicellulose can be seen in the second component, with the starting material (SP) near the alkaline treatment sample (AP) and bleaching I (B1P), indicating that the first steps were not efficient in removing hemicellulose. The loadings (Figure 3d) confirm this analysis, with the bands due to cellulose in the first component appearing around 1000 cm^−1^ and 1200 cm^−1^ and the bands due to hemicellulose in the second component appearing at around 1200 cm^−1^ and between 1600 cm^−1^ and 1700 cm^−1^ being those mainly responsible for the separation seen in the score plot.

#### 3.2.2. Powder X-ray Diffraction

**Figure 4:**
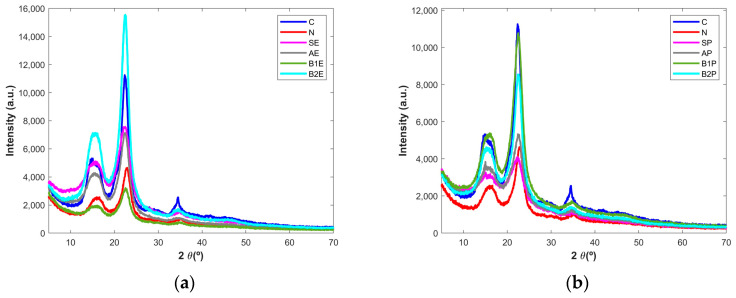
Powder X-ray diffractograms of the (**a**) eucalyptus and (**b**) pine tree wood samples after the different chemical treatment stages. C—microcrystalline cellulose; N—industrial paper pulp; S—starting materials; A—Alkaline treatment; B1—bleaching step I; B2—bleaching step II; E and P—eucalyptus-derived and pine tree-derived samples, respectively.

Powder X-ray diffraction analysis was performed to evaluate the crystallinity of the wood samples after different chemical treatment stages, as cellulose may have a crystalline or amorphous structure [13,31]. The diffraction patterns obtained for the starting materials, the alkaline-treated and bleached samples, and the commercially microcrystalline cellulose and paper pulp are shown in Figure 4a,b for the eucalyptus and pine tree samples, respectively. Three peaks can be observed in the powder X-ray diffractograms of all samples: a main peak at 2 Ɵ = 22.5°, a smaller peak at 2 Ɵ = 15.5°, and a more discrete peak at 2 Ɵ = 34.5°. The last signal was clearly present in the microcrystalline cellulose and was mainly observed in the wood fractions obtained after the second step of bleaching treatment (B2E and B2P). This peak is present in the other samples as well, though it generally presents lower intensities. These peaks are typical of the cellulose structure [14,28]. The samples’ amorphization can be seen by the formation of bands instead of well-defined peaks. For example, the sharp peak at 34.5° in the microcrystalline cellulose diffractogram is a broad band in the other diffractograms, showing that these samples are less crystalline than the microcrystalline cellulose. [2,3,11,13]. Additionally, the peaks became more defined upon chemical treatments, a fact that is more evident for pine tree-derived samples, meaning that the crystallinity increased. Nevertheless, in the last step of the extraction process both B2E and B2P present X-ray diffraction patterns closely related to the pure cellulose, pointing out the success of the extraction process as concerns the obtaining of a fraction with structural features similar to that characteristic of the commercially available cellulose and the diffractogram of paper pulp.

#### 3.2.3. Thermogravimetric Analysis

**Figure 5:**
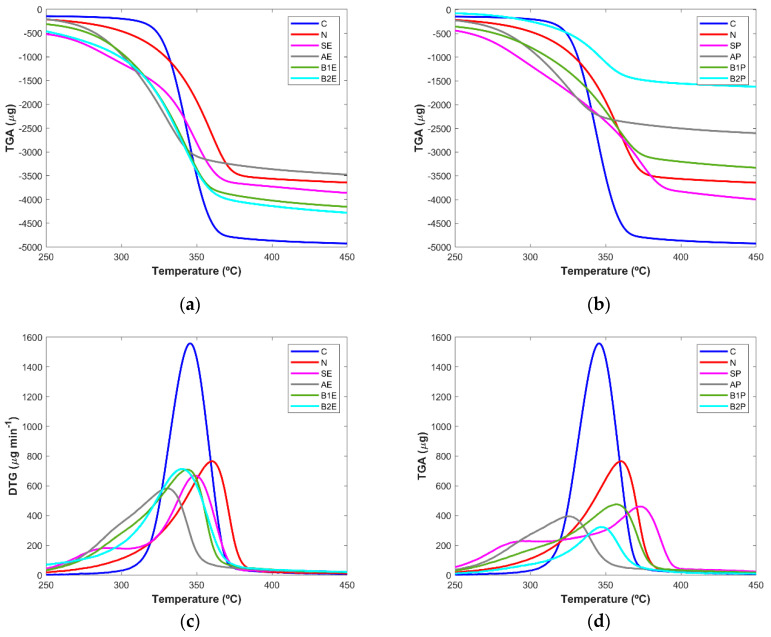
TGA thermograms (**a**,**b**) and DTG curves (**c**,**d**) of eucalyptus (**a**,**c**) and pine tree (**b**,**d**) wood extracts.

The purity of samples obtained from the microwave extraction in terms of cellulose content was analyzed based on the changes in the thermal behavior of the samples from the entire process as well as the two controls (C, N). The TGA curves depicted in Figure 5a,b show the differences in the thermal stability of the sample; based on this analysis, it was possible to infer the efficacy of each step. TGA data revealed that cellulose degradation began around 315 °C, with the sample with the higher rate of degradation reaching around 90% of weight loss for a temperature value up to 450 °C. The reference sample obtained from the paper company started to lose mass at approximately 290 °C until 378 °C, when it reached the maximum percentage of decomposition around 85%. The starting materials from eucalyptus (SE) and pine tree (SP) wood presented a slow rate of mass loss, starting from 260/350 °C until 380/400 °C, respectively. These samples showed larger decay in terms of weight loss at higher temperature values than was registered for the extracts of the following steps and for cellulose control, which was more evident in eucalyptus samples.

The extracts obtained after alkaline treatment (AE and AP) showed a similar thermal stability profile, and the weight loss was verified for a lower temperature range from 260 °C to 365 °C. This can be explained by the presence of different wood components, namely, hemicellulose and lignin, in agreement with the results obtained by Ndruru et al. [12], as these components start their decomposition at lower temperature values than those described for pure cellulose.

The B1E and B2E products showed similar decomposition profiles; larger differences were verified for the B1P and B2P samples, in which the extract resulting from the last step of the methodology lost a small weight, being almost totally degraded (~83%) at 370 °C.

Other studies performed by Morán and co-workers [28] have demonstrated that cellulose starts its decomposition at higher temperature values (~315 °C), while hemicellulose and lignin start to lose weight at lower values of approximately 220 °C and 200 °C, respectively. This information agrees with the results reported here, as in general the extracts showed decomposition in a temperature range lower than that found for pure cellulose.

From the derivative thermogravimetric (DTG) curves (Figure 5c,d), a more evident evaluation of the success of the extraction steps can be inferred. It was possible to visualize a sharp and well-defined peak for the pure cellulose (345 °C), while the curves from the starting materials and alkaline samples seem to comprise two bands at approximately 283/287 °C and 348/373 °C (SE/SP) or 293/292 °C and 330/325 °C (AE/AP), respectively, for each step. The presence of these two bands is probably due to the cellulose and other wood components. Interestingly, the DTG curves clearly show that these extra components were successfully removed during the following steps, as the curves for B2E and B2P do not present a defined second peak (Figure 5c, d). For the eucalyptus samples, no major differences (~3 °C) were found between B1E (344 °C) and B2E (Figure 5c), indicating that the most relevant step for the removal of hemicellulose and lignin in this type of wood was the first bleaching treatment. A small difference of approximately 10 °C was found for the band’s correspondence to B1P and B2P extract (Figure 5d), which appeared at 357 and 347 °C, respectively, suggesting that the last extraction step still had a slight contribution to the removal of non-cellulosic components from the pine tree samples. This conclusion was already obtained by the PCA analysis of the FTIR spectra.

The obtained results are in good agreement with those reported for the thermogravimetric analysis of products obtained by other cellulose extraction methodologies [2,3,13,28]. Globally, TGA studies have demonstrated a clear evolution in cellulose isolation as the extraction steps proceed, evidenced by the great similarity between the DTG curves from the bleaching II step and the pure cellulose.

#### 3.2.4. Differential Scanning Calorimetry

**Figure 6:**
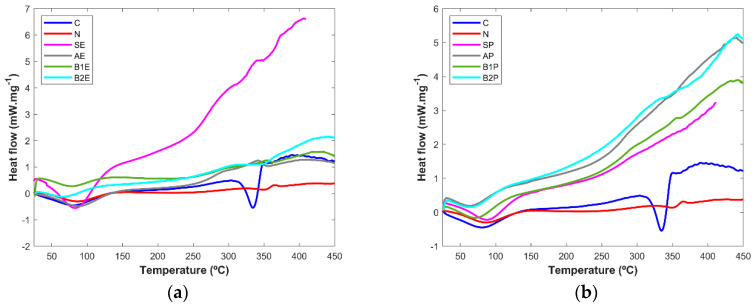
DSC curve of (**a**) eucalyptus and (**b**) pine tree wood samples.

DSC was considered as another technique to fully characterize the extraction fractions obtained in each step and compare them with the pure microcrystalline cellulose and the sample from paper industry processing (Figure 6). In all samples, the results show a first endothermic event at around 100 °C in both pine and eucalyptus extracts, which is due to water evaporation [3,28]. Melting at approximately 300 °C in the cellulose control (C) revealed the higher degree of crystallinity of this sample. In particular, the starting material from eucalyptus wood (SE) showed three endothermic events at around 250, 300, and 350 °C, which could be the melting of the main components of the wood, cellulose, hemicellulose, and lignin [3,28]. All the other samples, including the one obtained from the industrial processing (N), show extremely low crystallinity, and the endothermic peak due to cellulose melting cannot be seen (pine extracts) or is very small (eucalyptus samples). The lack of crystallinity shown in the DSC thermograms is in accordance with the XRPD results.

## 4. Conclusions

The results of the present work allow the contemplation of a new, simple, and sustainable microwave-assisted protocol for cellulose extraction from eucalyptus and pine tree wood waste.

FTIR studies, particularly PCA data, corroborate the conclusions from TGA studies and XRPD diffractograms confirming the presence of cellulose in all extracts. It can be concluded that the removal of hemicellulose is more efficient in the last beaching step. The analysis of DTG curves shows that the starting materials and the samples from the first extraction step are composed of more than one component. This fact indicates the benefit of the subsequent extraction steps, as the final products, especially those from bleaching II, are closer to cellulose. This is more evident for pine samples. The results confirm that for pine, which is a softwood, the delignification process is more difficult than for eucalyptus, which is a hardwood, as in eucalyptus products hemicellulose is more easily removed in the earlier extraction steps. Nevertheless, after all the steps considered in the protocol, it was possible to obtain cellulose in a comparable way for both types of samples. At the end of the process, it was possible to conclude that hemicellulose was totally removed from the starting materials, while lignin was partially removed.

The overall results reveal the applicability of this new approach as a sustainable strategy based on green methodologies such as MAE and mild extraction/purification conditions, making it encouraging to pursue the full quantitative characterization of the process. The established protocol enables the obtention of cellulose from softwood and hardwood, particularly from pine and eucalyptus waste products, and offers the possibility for further testing as a valuable tool to extract this biopolymer from other natural sources, such as algae and many diverse types of plant feedstocks. Moreover, the fact that the methodology was exclusively developed using multimode microwave equipment offers a great opportunity to scale up the process for eventual consideration by industry.

## Data Availability

Data are contained within the article.
